# Genome-wide identification and expression analysis of the *SWEET* gene family in daylily (*Hemerocallis fulva*) and functional analysis of *HfSWEET17* in response to cold stress

**DOI:** 10.1186/s12870-022-03609-6

**Published:** 2022-04-25

**Authors:** Dong-Mei Huang, Ying Chen, Xiang Liu, Di-An Ni, Lu Bai, Qiao-Ping Qin

**Affiliations:** School of Ecological Technology and Engineering, Shanghai Institute of Technology, Shanghai, 201418 China

**Keywords:** Daylily, SWEET, Expression patterns, Cold stress, Functional analysis

## Abstract

**Background:**

The Sugars Will Eventually be Exported Transporters (*SWEET*s) are a newly discovered family of sugar transporters whose members exist in a variety of organisms and are highly conserved. *SWEET*s have been reported to be involved in the growth and development of many plants, but little is known about *SWEET*s in daylily (*Hemerocallis fulva*), an important perennial ornamental flower.

**Results:**

In this study, 19 daylily *SWEET*s were identified and named based on their homologous genes in *Arabidopsis* and rice. Phylogenetic analysis classified these *HfSWEET*s into four clades (Clades I to IV). The conserved motifs and gene structures showed that the *HfSWEET*s were very conservative during evolution. Chromosomal localization and synteny analysis found that *HfSWEET*s were unevenly distributed on 11 chromosomes, and there were five pairs of segmentally duplicated events and one pair of tandem duplication events. The expression patterns of the 19 *HfSWEET*s showed that the expression patterns of most *HfSWEET*s in different tissues were related to corresponding clades, and most *HfSWEET*s were up-regulated under low temperatures. Furthermore, *HfSWEET17* was overexpressed in tobacco, and the cold resistance of transgenic plants was much higher than that of wild-type tobacco.

**Conclusion:**

This study identified the *SWEET* gene family in daylily at the genome-wide level. Most of the 19 *HfSWEET*s were expressed differently in different tissues and under low temperatures. Overexpression further suggests that *HfSWEET17* participates in daylily low-temperature response. The results of this study provide a basis for further functional analysis of the *SWEET* family in daylily.

**Supplementary Information:**

The online version contains supplementary material available at 10.1186/s12870-022-03609-6.

## Introduction

As the substrate of carbon and energy metabolism, sugar provides energy sources for plant growth and development, and promotes many physiological processes in plants, such as seed germination, photosynthesis, and flowering [1–3]. Sugar is also involved in host–pathogen interactions and various abiotic stress responses in plants [4–6]. However, sugar cannot independently cross the plant biomembrane system and requires the assistance of the transport function of the corresponding sugar transporter [7]. At present, three eukaryotic sugar transporter families, SUTs (sucrose transporters), MSTs (monosaccharide transporters), and SWEET, have been identified in plants [8–10]. Among them, SWEET protein is largely pH-independent and acts as a bidirectional transmembrane transporter of sugar along the concentration gradient [11, 12]. SWEETs can selectively transport monosaccharides or disaccharides within cells or across the plasma membrane [13], and are widely found in prokaryotes, plants, humans, and other animals [14, 15]. The typical structures of eukaryotic SWEET proteins consist of seven transmembrane helices, harboring two MtN3/saliva domains that are also known as PQ-loop-repeat [16]. However, the SWEET protein in prokaryotes contains only three transmembrane helical proteins and one MtN3/saliva domain [17]. This difference may indicate that the eukaryotic SWEET protein evolved by replicating and fusing the basic 3-TM unit present in the prokaryotic semi-SWEET protein [18].

The first identified plant SWEET transporter is AtSWEET1, which acts as a single glucose transporter and is involved in flower development by supplying nutrients to the gametophyte or nectaries [13]. To date, the genome-wide identification and analysis of the *SWEET* gene family have been reported in a variety of plant species, such as *Arabidopsis thaliana*, rice (*Oryza sativa*), *Gossypium hirsutum*, *Sorghum bicolor*, *Litchi chinensis*, and *Glycine max *[13, 15, 16, 18–20]. Numerous studies have shown that *SWEET*s are involved in multiple biological processes, such as reproductive development, seed and fruit development, gibberellin modulation, disease resistance, and abiotic stress responses [21–24]. *AtSWEET4* overexpression lines have higher plant heights, while mutant lines show shorter heights, as well as lower fructose and glucose contents in leaves [25]. Mutants of both maize *ZmSWEET4c* and its rice ortholog *OsSWEET4* are defective in seed filling, suggesting that these genes play important roles in seed development [23]. *OsSWEET3a* in rice affects plant growth and development through gibberellin-mediated responses, both knockout and overexpression lines of *OsSWEET3a* show defects in germination and early shoot development [26]. Overexpression of *IbSWEET10* increases tolerance to *Fusarium oxysporum* infection [27], and overexpression lines of *AtSWEET16* show higher freezing tolerance [28].

*SWEET17*, a Clade IV member, plays an important role in plant development and abiotic stress response. *AtSWEET17* is a vacuolar fructose transporter that participates in the regulation of fructose levels and controls leaf fructose content [29], and plays a key role in exporting fructose from leaf vacuoles. For example, the fructose content in the leaves of *AtSWEET17* overexpression lines decreased by 80% under cold stress [30]. *AtSWEET17* expression was found to be significantly elevated during lateral root growth and under drought conditions, and *SWEET17* knock-out mutants exhibited reduced lateral root growth and decreased expression of lateral root development-related transcription factors during drought stress, and impaired drought tolerance in the plants themselves [31]. The overexpression of *DsSWEET17*, a homolog of *AtSWEET17*, promoted *Arabidopsis* root length, fresh weight, and growth rate by affecting sugar metabolism and conferred multiple abiotic stress tolerances to plants [32].

Daylily (*Hemerocallis fulva*) is an herbaceous perennial plant, with edible, medicinal, and ornamental value that is widely cultivated worldwide. In recent years, daylily has been the focus of biological research, and a growing number of reports have explored daylily molecular mechanisms and gene function analysis [33–36]. A previous study by this research group analyzed the characteristics of the *HfSWEET2a* gene in daylily and its expression level changes under low temperatures [37]. There have been no other reports on *SWEET* genes in daylily. In the current study, a whole genome-wide analysis was performed to identify *SWEET*s in daylily and analyze their phylogenetic relationships, gene structures, chromosomal localization, conserved motifs, and domains in detail. In addition, the expression characteristics of daylily *SWEET* gene family members in different tissues and under low temperatures were investigated. The *HfSWEET17* gene was then transformed into tobacco through an *Agrobacterium*-mediated method to investigate its function. The results of this study provide data that could aid in elucidating the function and cold responses of *SWEET* genes in daylily.

## Results

### Identification of the daylily *SWEET* gene family

A total of 38 *SWEET* gene sequences were retrieved from the daylily genome Through the screening, 19 of these sequences were retained and named as *HfSWEET1*–*HfSWEET17* (GenBank accession Nos. OM264165–OM264183, Additional file 1: Table S1) according to their identity percentage with *Arabidopsis AtSWEET*s and rice *OsSWEET*s. Gene characteristics, including the complete open reading frames (ORFs), number of amino acids (AA), and molecular weight (MW), isoelectric point (pI) were analyzed (Table 1). The results showed that the ORFs of the 19 *HfSWEET*s ranged from 699 to 900 bp in length, encoding proteins 232 aa to 299 aa. The HfSWEET7 protein had the smallest MW at 25.764 kDa, and the highest MW was found in HfSWEET16 at 32.976 kDa. The pI ranged from 4.74 (HfSWEET17) to 9.64 (HfSWEET16), which indicated that most of the HfSWEET proteins were basic proteins. The instability index ranged from 27.15 (HfSWEET1b) to 48.05 (HfSWEET12), revealing that HfSWEET proteins consisted of both stable and unstable proteins. All of the HfSWEET proteins were hydrophobic proteins (grand average of hydropathicity, GRAVY > 0). These results indicated that the basic properties of the proteins encoded by members of the daylily *HfSWEET* gene family were different.

**Table 1:** Information about daylily SWEET genes

Gene name	ORF length (bp)	AA (aa)	MW(kDa)	pI	II	AI	GRAVY	THM	MtN3/saliva (PQ-loop repeat) domain position
HfSWEET1a	735	244	27.162	9.26	40.21	111.80	0.656	7	7–95, 132–214
HfSWEET1b	762	253	28.351	9.01	27.15	105.49	0.519	7	7–95, 132–214
HfSWEET2a	699	232	25.908	8.81	42.55	124.78	1.018	7	18–100, 138–218
HfSWEET3b	714	237	26.322	9.44	38.27	117.22	0.700	7	7–98, 132–217
HfSWEET4a	774	257	28.370	9.30	28.83	127.35	0.800	6	10–95, 133–217
HfSWEET4b	735	244	26.993	8.89	37.65	130.82	0.942	7	10–97, 133–217
HfSWEET4c	735	244	26.959	8.95	34.37	132.87	0.941	7	10–98, 133–217
HfSWEET5	714	237	25.932	8.63	31.03	131.52	0.891	7	11–96, 134–213
HfSWEET6a	714	237	26.235	9.21	44.96	133.59	0.957	7	9–98, 133–217
HfSWEET6b	840	279	30.460	9.03	35.24	118.14	0.669	7	11–96, 133–217
HfSWEET7	711	236	25.764	9.22	36.30	134.49	1.106	7	10–95, 133–213
HfSWEET12	786	261	29.196	8.94	48.05	122.53	0.721	7	14–98, 132–218
HfSWEET13a	825	274	30.706	9.20	29.62	118.43	0.667	7	12–99, 133–214
HfSWEET13b	870	289	32.278	5.74	34.67	116.99	0.516	7	12–82, 134–215
HfSWEET14a	861	286	32.412	8.80	33.51	123.36	0.593	7	12–99, 133–215
HfSWEET14b	861	286	32.276	8.80	31.49	122.38	0.596	7	12–99, 133–215
HfSWEET15	870	289	32.116	5.49	38.42	121.45	0.724	7	12–98, 134–215
HfSWEET16	900	299	32.976	9.64	34.03	112.04	0.442	7	7–91,128–211
HfSWEET17	723	240	26.934	4.74	43.23	119.67	0.730	7	7–90,128–212

*AA* Number of Amino Acids, *MW* Molecular Weight(kDa), *pI* Isoelectric Point, *II* Instability Index, *AI* Aliphatic Index, *GRAVY* Grand Average of hydropathicity, *THM* Prediction of the number of Transmembrane Helix

### Phylogenetic analysis of the *HfSWEET* family

**Fig. 1:**
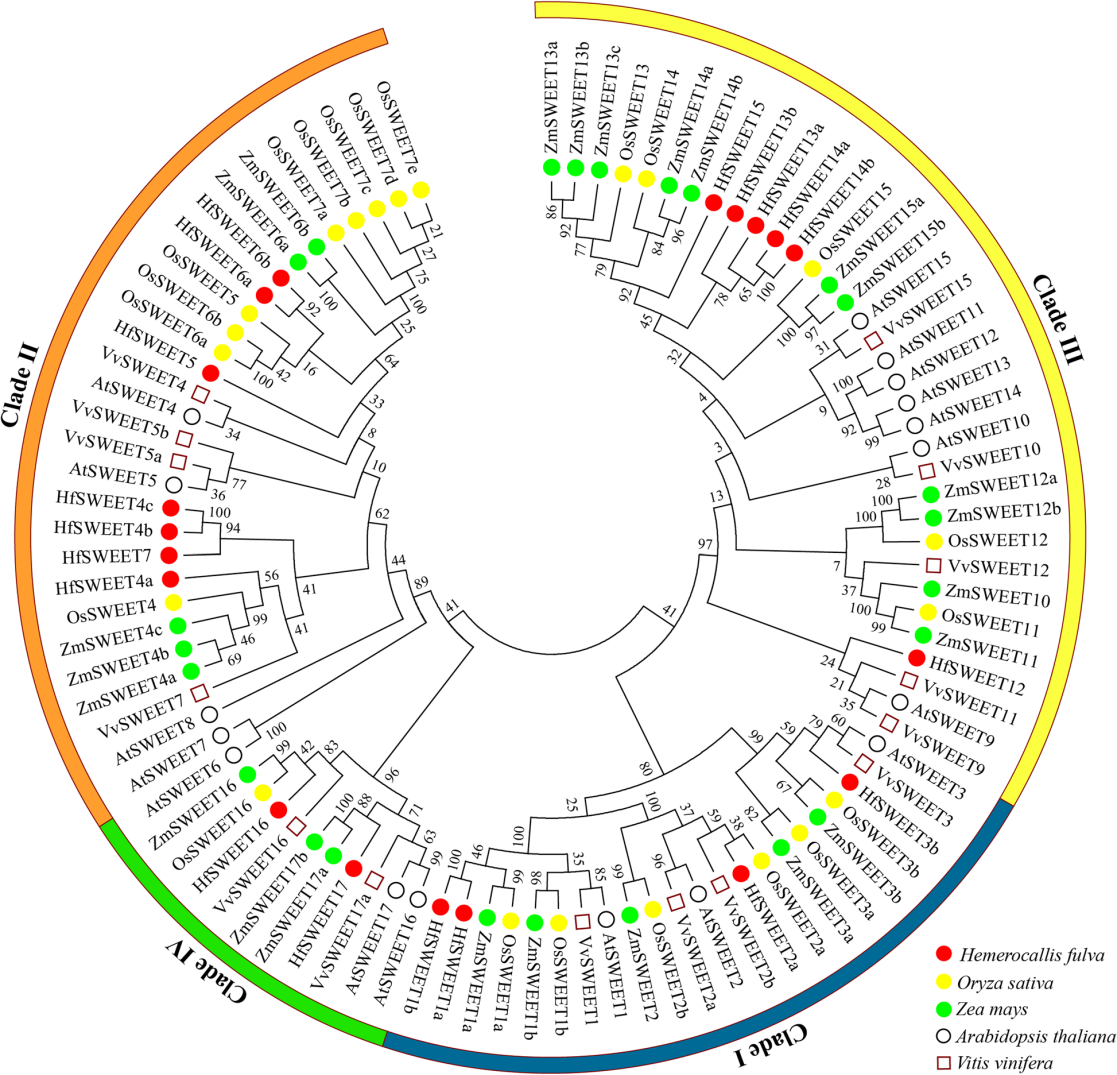
Phylogenetic tree of SWEETs from daylily and other plants. The protein sequences of the 96 SWEETs from daylily, rice, *Zea mays*, *Arabidopsis*, and *Vitis vinifera* were aligned by Clustal Omega, and the phylogenetic tree was constructed by MEGA7.0 using the neighbor-joining method with 1000 bootstrap replicates. SWEETs of daylily, rice, *Zea mays*, *Arabidopsis*, and *Vitis vinifera* were prefixed with Hf, Os, Zm, At, and Vv, respectively

To investigate the evolutionary relationships among HfSWEETs and SWEET proteins from other plants (Additional file 2: Table S2), a neighbor-joining phylogenetic tree was constructed using MEGA 7.0. The results showed that the HfSWEET proteins were divided into four clades: Clades I, II, III, and IV (Fig. 1). The largest was Clade II, which consisted of seven HfSWEET proteins (HfSWEET4a/4b/4c/5/6a/6b/7); the second-largest was Clade III, which contained six HfSWEET proteins (HfSWEET12/13a/13b/14a/14b/15); Clade I contained four HfSWEET proteins (HfSWEET1a/1b/2a/3b); and Clade IV was the smallest, containing only two HfSWEET proteins (HfSWEET16/17). Compared with dicotyledons, the similarity between SWEET proteins in daylily and those in monocotyledons was higher, indicating that SWEET proteins in daylily were more closely related to monocotyledons than to dicotyledons.

To obtain more detailed information concerning the HfSWEET17 proteins, this study performed multiple sequence alignments of the HfSWEET17 and SWEET17 proteins from 24 other plants. The results showed that the conserved domains of HfSWEET17 shared high similarities with other plants (Fig. S1 in Additional file 3). The results of phylogenetic analysis showed that the monocotyledonous and dicotyledonous SWEET17 proteins were clustered into two different categories, and the phylogenetic relationship between HfSWEET17 and SWEET17 from *Ananas comosus* was the closet (Fig. S2 in Additional file 3).

### Conserved motifs and conserved domains analyses of HfSWEETs

**Fig. 2:**
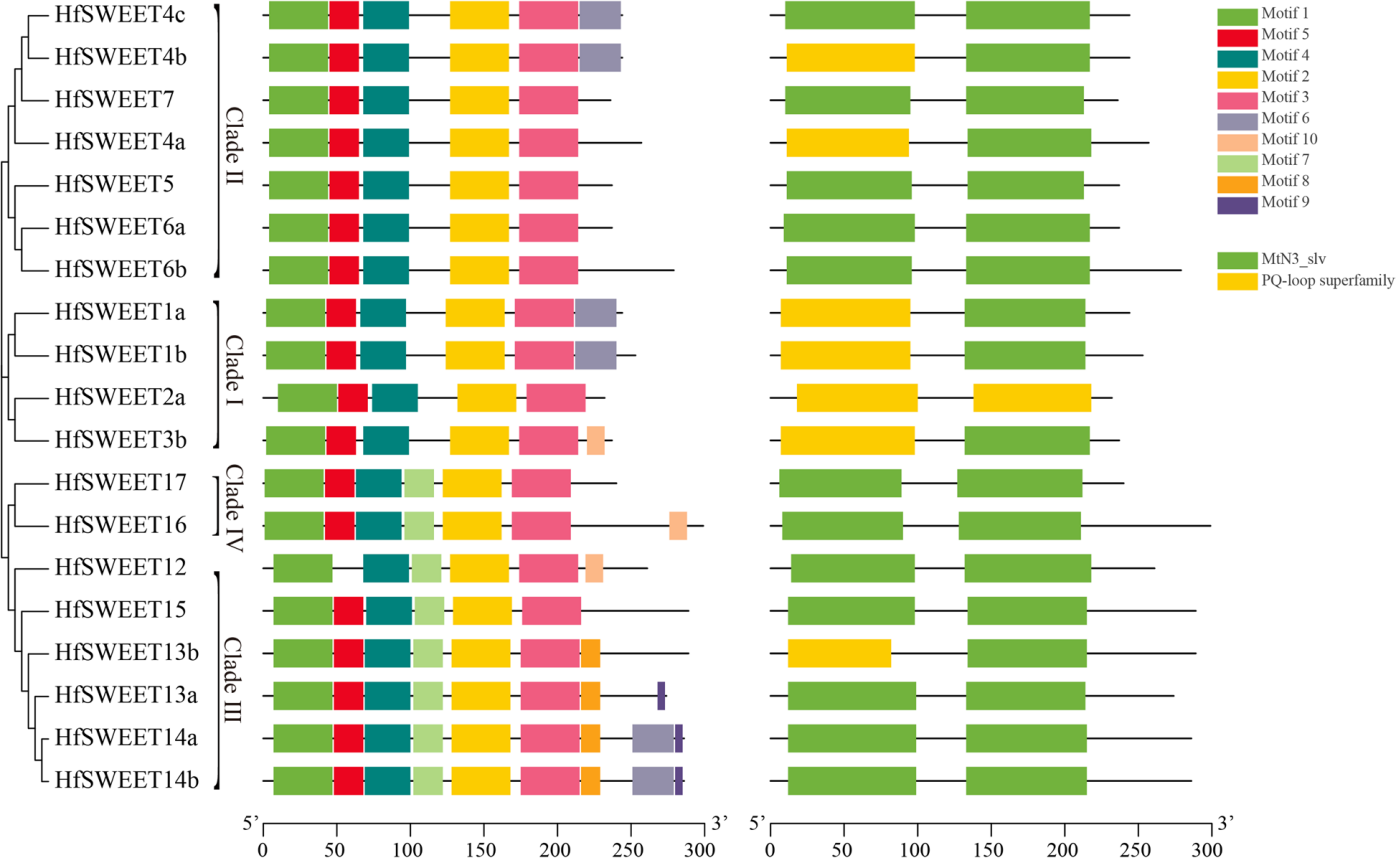
Phylogenetic relationships, conserved motifs and conserved domain analyses of HfSWEET proteins. **A** The neighbor-joining phylogenetic tree of putative HfSWEET proteins was constructed by MEGA7 with 1000 bootstrap replicates. The classified Clades of I, II, III, and IV are marked. **B** The motif compositions of HfSWEET proteins. Ten motifs are displayed in different colored rectangles. **C** The domain composition of HfSWEETs. Green rectangles represent the MtN3/saliva domain and yellow rectangles represent the PQ-loop domain

The conserved motifs and conserved domains were analyzed to further understand the characteristics of HfSWEETs. The results of conserved motif analysis showed that a total of 10 motifs were identified and named Motifs 1–10 (Fig. 2). Motifs 1 to 5 were detected in all HfSWEET proteins except HfSWEET12, which lacked Motif 5. Motif 6 was detected in two members each of Clades I, II, and III, but not in any member of Clade IV. Motif 7 was detected in all the members of Clade III and Clade IV. Motifs 8 and 9 were only detected in some members of Clade III. Motif 10 was only detected in the HfSWEET3b, HfSWEET12, and HfSWEET16 proteins.

To obtain more detailed information about HfSWEETs, a multiple sequence alignment of the HfSWEET protein sequences was conducted. The results showed that the majority of the HfSWEETs contained seven transmembrane domains, and only HfSWEET4a contained six transmembrane domains (Fig. S3 in Additional file 3). Additionally, the protein sequences of the HfSWEET family members were relatively conserved, and all the HfSWEET proteins harbored two MtN3/saliva domains (CDD accession No. pfam03083) or the PQ-loop superfamily (CDD accession No. cl21610) at similar positions (Fig. S3 in Additional file 3 and Fig. 2). These MtN3/saliva domains ranged from 70 to 91 aa, and most of them were approximately 85 aa in length. The positions of the MtN3/saliva domains in the protein are shown in Table 1.

### Gene structure analysis of *HfSWEETs*

**Fig. 3:**
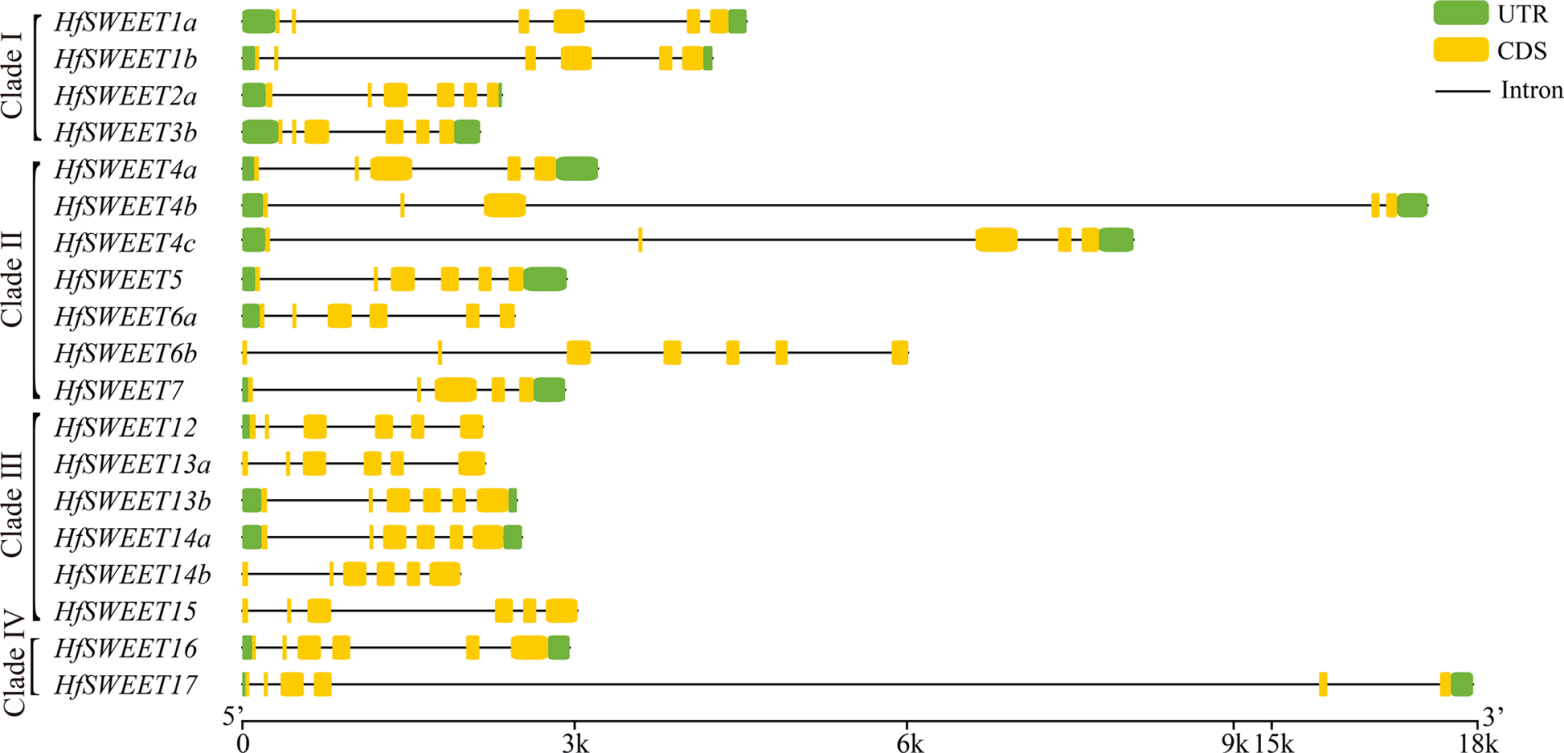
Gene structures of *HfSWEET*s from daylily. The exon–intron structures were analyzed by GSDS (http://gsds.cbi.pku.edu.ch). Exons are represented by green boxes, the upstream and downstream are represented by blue boxes, and introns are represented by black lines

To elucidate the structural characteristics of *SWEET*s in daylily, the exon–intron organization was analyzed. The result showed that five or six exons existed in most *HfSWEET*s (Fig. 3). The *HfSWEET*s in Clades I, III, and IV all contained six exons; the majority of *HfSWEET*s in Clade II contained five exons, *HfSWEET5* and *HfSWEET6a* contained six exons, and *HfSWEET7* contained seven exons. In general, the intron lengths of *HfSWEET*s in Clades II and IV were longer than those in Clades I and III. These results revealed that *HfSWEET*s in the same clade shared a similar gene structure.

### Chromosomal localization and synteny analysis of *HfSWEETs*

**Fig. 4:**
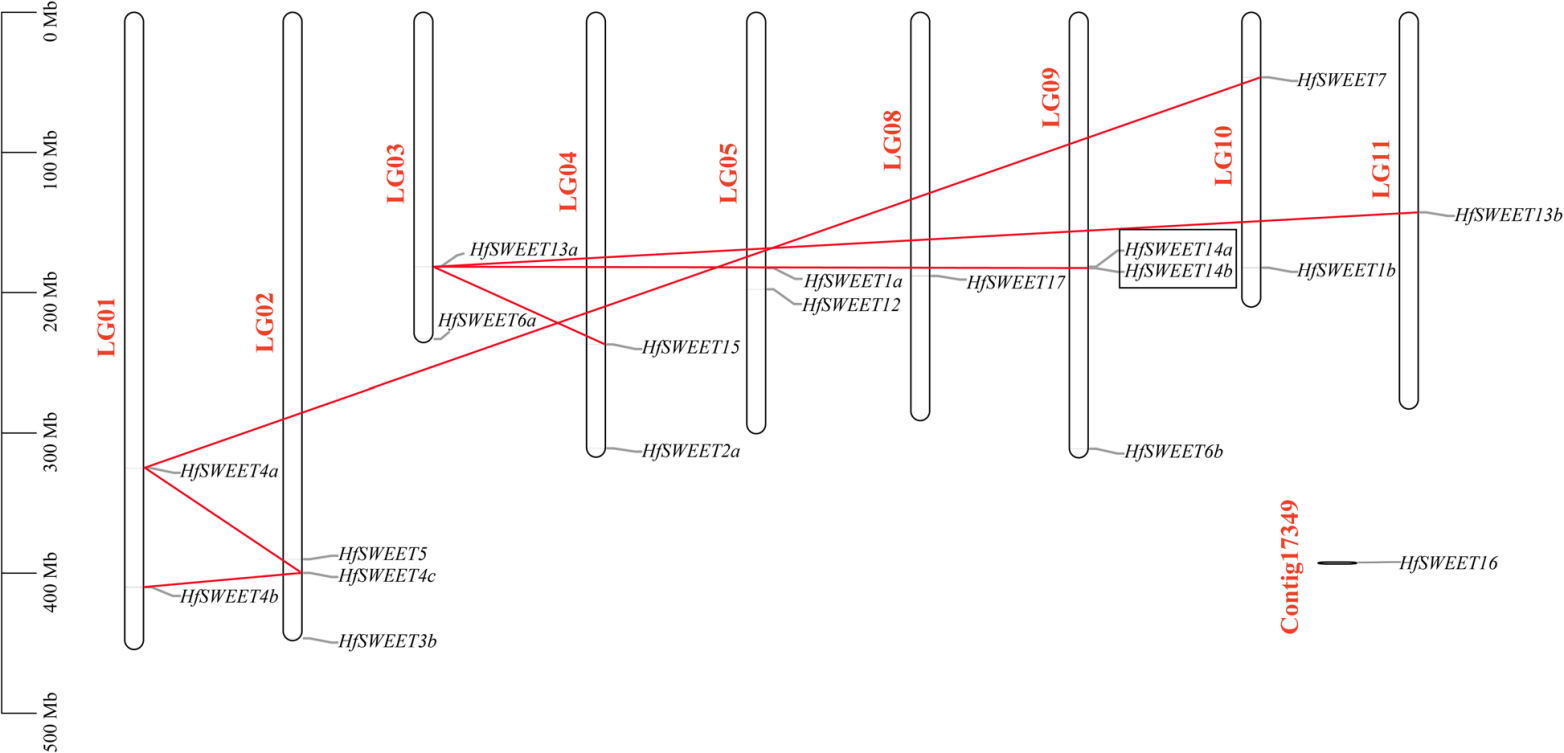
Locations and duplications of *HfSWEET*s on daylily chromosomes. The chromosome locations of *HfSWEET*s are indicated by short grey lines. The red lines indicate segmentally duplicated genes, and the tandemly duplicated genes are boxed

According to the gene loci information, the 18 *HfSWEET*s were unevenly distributed on 11 daylily chromosomes. The detailed chromosomal locations are shown in Fig. 4. However, *HfSWEET16* was distributed on a scaffold whose exact locations on the chromosome were not determined. Chromosomes 2 and 9 had the largest number of *HfSWEET*s (three genes), followed by chromosomes 1, 3, 4, 5, and 10 (two genes on each chromosome), and the minimum number was found on chromosomes 8 and 11 (one gene). Except for *HfSWEET7*, the other *HfSWEET*s were located in the middle or lower part of the chromosomes.

**Fig. 5:**
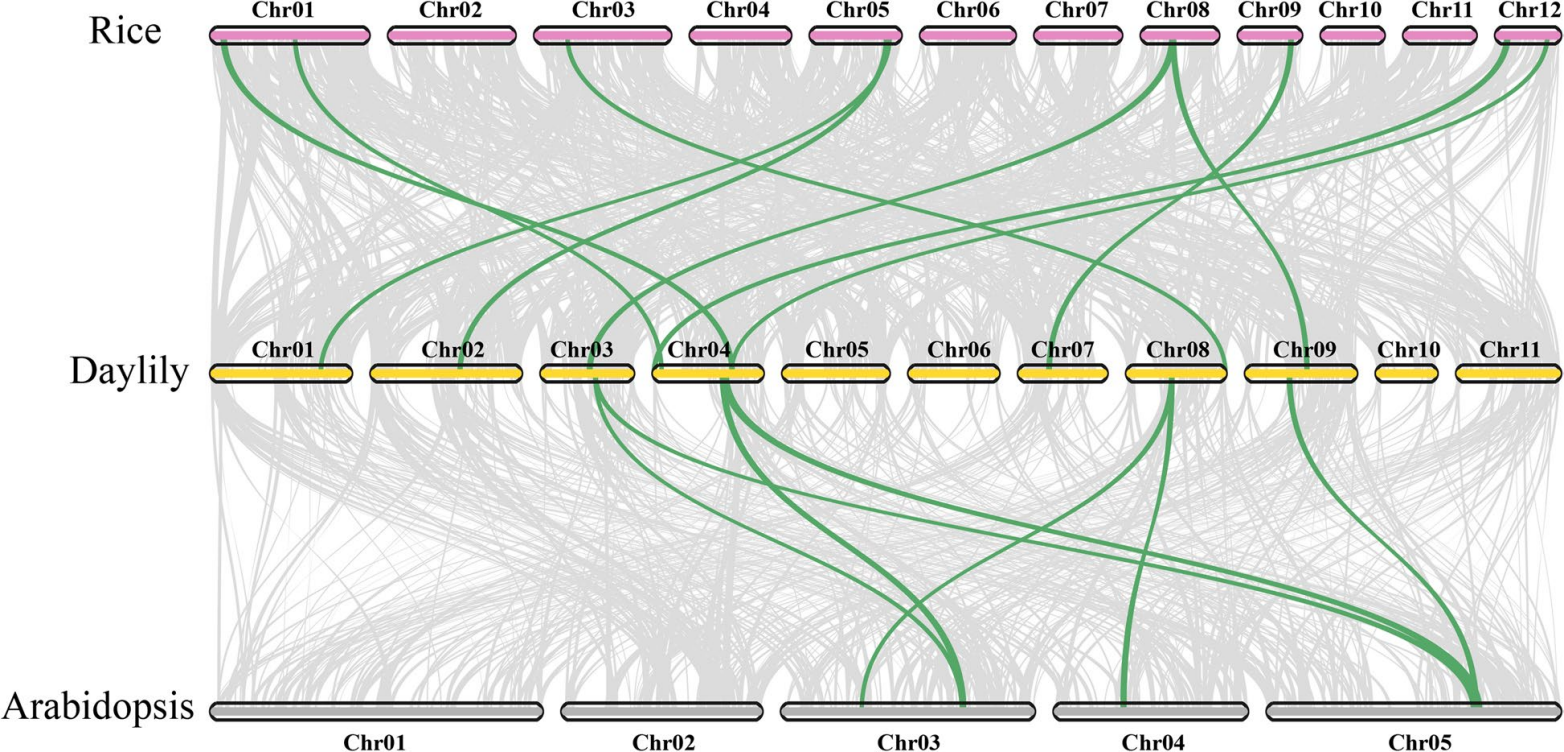
The collinearity relationship between the daylily, *Arabidopsis*, and rice genomes. The collinear relationships between the daylily, *Arabidopsis*, and rice genomes are shown on the chromosome maps. The gray line represents the collinearity among all members, and the green line represents the collinearity among the members of the SWEET family

The results of collinearity analysis showed that there were five pairs of segmental duplication events in daylily *HfSWEET*s. The most frequently duplicated gene was *HfSWEET13a*, which was duplicated three times, corresponding to *HfSWEET13b*, *HfSWEET14b*, and *HfSWEET15*. *HfSWEET4a/7* and *HfSWEET4a/4b/4c* might also have been generated by fragment duplication. In addition, *HfSWEET14a* and *HfSWEET14b* were clustered into tandem duplication events. These results indicated that some *HfSWEET*s were probably generated by gene segmental or tandem duplication. The results of collinearity analysis between daylily and *Arabidopsis* and rice showed that seven *HfSWEET* homologous protein genes appeared in the last three chromosomes of *Arabidopsis* (Fig. 5), but nine *HfSWEET*s had corresponding paralogous genes on six chromosomes on rice. The relationship between daylily and rice is closer than that between daylily and *Arabidopsis*.

### Expression patterns of *HfSWEETs* in different tissues

**Fig. 6:**
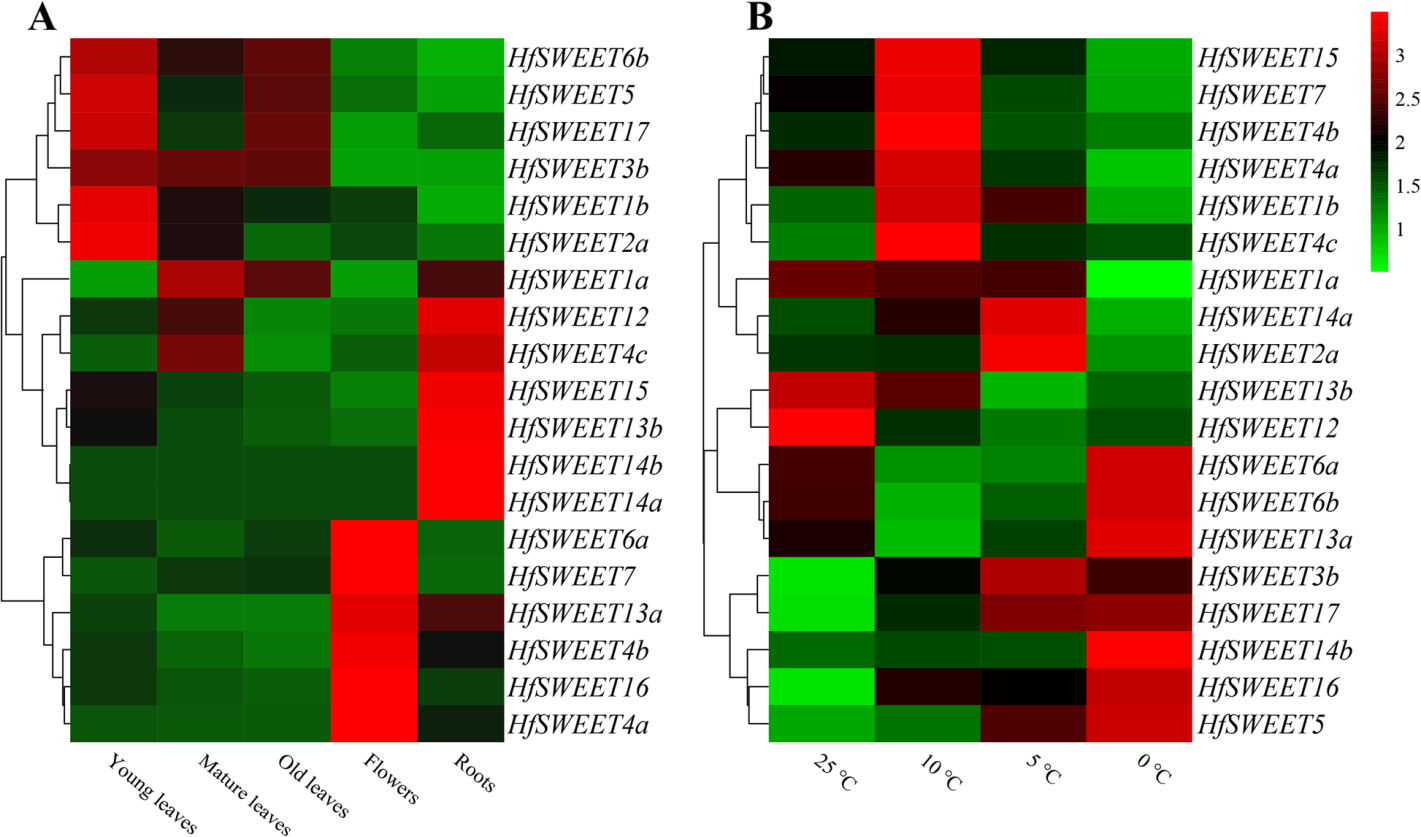
Expression patterns of *HfSWEET*s. **A** Expression patterns of *HfSWEET*s in different tissues, including young leaves, mature leaves, old leaves, flowers, and roots. **B** Expression patterns of *HfSWEET*s under different temperatures. Samples were collected from mature leaves. The control group (CK) was grown at 25 °C, while 10 °C, 5 °C and 0 °C were the low-temperature treatment groups. The colored bar represents the average of the relative expression levels. Statistical analysis was performed by SPSS 20 software, and a one-way analysis of variance (ANOVA) was performed for the relative expression of *HfSWEET*s in different tissues and response to low-temperature stress

To obtain insights into the physiological functions of the *HfSWEET*s, a real-time quantitative PCR (qRT-PCR) assay was performed to detect the expression patterns of 19 *HfSWEET*s in different tissues, including young leaves, mature leaves, old leaves, flowers, and roots. The results showed that the expression patterns of 19 *HfSWEET*s differed in daylily organs (Fig. 6A). The relative expression levels of the majority of *HfSWEET*s in young leaves were higher than those in mature leaves and old leaves. All of the *HfSWEET*s in Clade I (*HfSWEET1a*/*1b*/*2a*/*3b*) showed the highest relative expression in leaves. Most of the *HfSWEET*s in Clade II (*HfSWEET4a*/*4b*/*6a*/*7*) showed the highest relative expression in flowers, while *HfSWEET5* and *HfSWEET6b* had high expression levels in young leaves. Most of the *HfSWEET*s in Clade III (*HfSWEET12*/*13b*/*14a*/1*4b*/*15*) showed the highest relative expression in roots. However, the *HfSWEET13b*/*14a*/1*4b* genes were close to undetectable detected in the old leaves and flowers. In Clade IV, *HfSWEET17* showed higher relative expression in young leaves and roots, while *HfSWEET16* showed higher relative expression in flowers. In general, most *HfSWEET*s in the same clade shared similar expression patterns.

### Expression patterns of *HfSWEETs* in response to low-temperature stress

To further understand the physiological functions of the *HfSWEET*s in response to low-temperature stress, the expression patterns of *HfSWEET*s under different temperatures (25 °C as a control group (CK), 10 °C, 5 °C, and 0 °C as low-temperature treatments) were measured. The results showed that the expression patterns were different among the 19 *HfSWEET*s (Fig. 6B). Compared with the CK, with the decrease in temperature, the relative expression levels of nine *HfSWEET*s increased first and then decreased, but three *HfSWEET*s showed a contrary expression trend. The relative expression levels of five *HfSWEET*s (*HfSWEET3b*/*5*/*14b*/*16*/*17)* were higher than the CK in all low-temperature treatments. Among them, the expression levels of *HfSWEET5* and *HfSWEET17* rose steadily as the temperature dropped. However, three *HfSWEET*s (*HfSWEET1a*/*12*/*13b*) were lower than CK at all lower temperatures, and the expression level of *HfSWEET1a* gradually decreased with the decrease of temperature. In general, the relative expression level of the majority *HfSWEET*s was up-regulated by the low-temperature treatments, and most of these genes exhibited the highest expression at 10 °C or 0 °C, 1.43–57.95 times more than the expression found in the CK.

### Subcellular localization of HfSWEET17

**Fig. 7:**
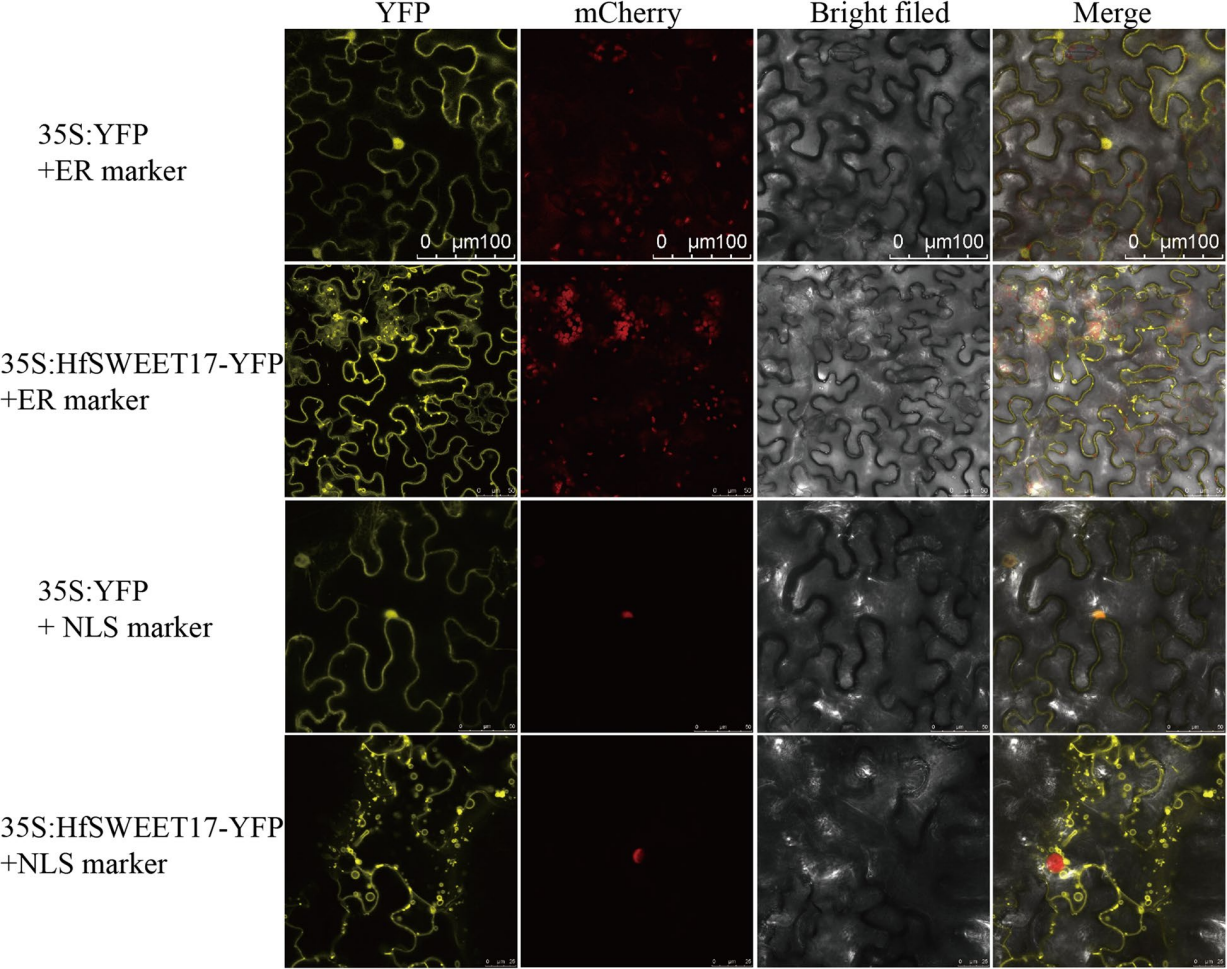
Subcellular localization of HfSWEET17 fusion protein. The 35:HfSWEET17-YFP was generated by the insertion of the open reading frame (ORF) of *HfSWEET17* into the pC131-YFP vector framework, and transformed into GV3101. Organelle markers, including endoplasmic reticulum (ER) marker HDEL-mCherry, and nuclear localization sequence (NLS) marker pBin-NLS-mCherry, were used in co-transformation experiments with the 35S:HfSWEET17-GFP to determine its subcellular localization. Confocal laser microscopy scanning was carried out 48 h after dark culture. Representative images are shown. Leaves transformed with the 35S:YFP vector alone were used as a control

The subcellular localization of HfSWEET17 was studied to evaluate where it functioned. HfSWEET17 protein was transiently expressed as translational yellow fluorescent protein (YFP) fusion proteins in tobacco leaf epidermal cells. Confocal scanning results showed that 35S:HfSWEET17-YFP was mainly present in the tonoplast and plasma membrane. Signals in the endoplasmic reticulum (ER) were detected, but not in the nucleus (Fig. 7).

### Ectopic expression of *HfSWEET17* in tobacco

**Fig. 8:**
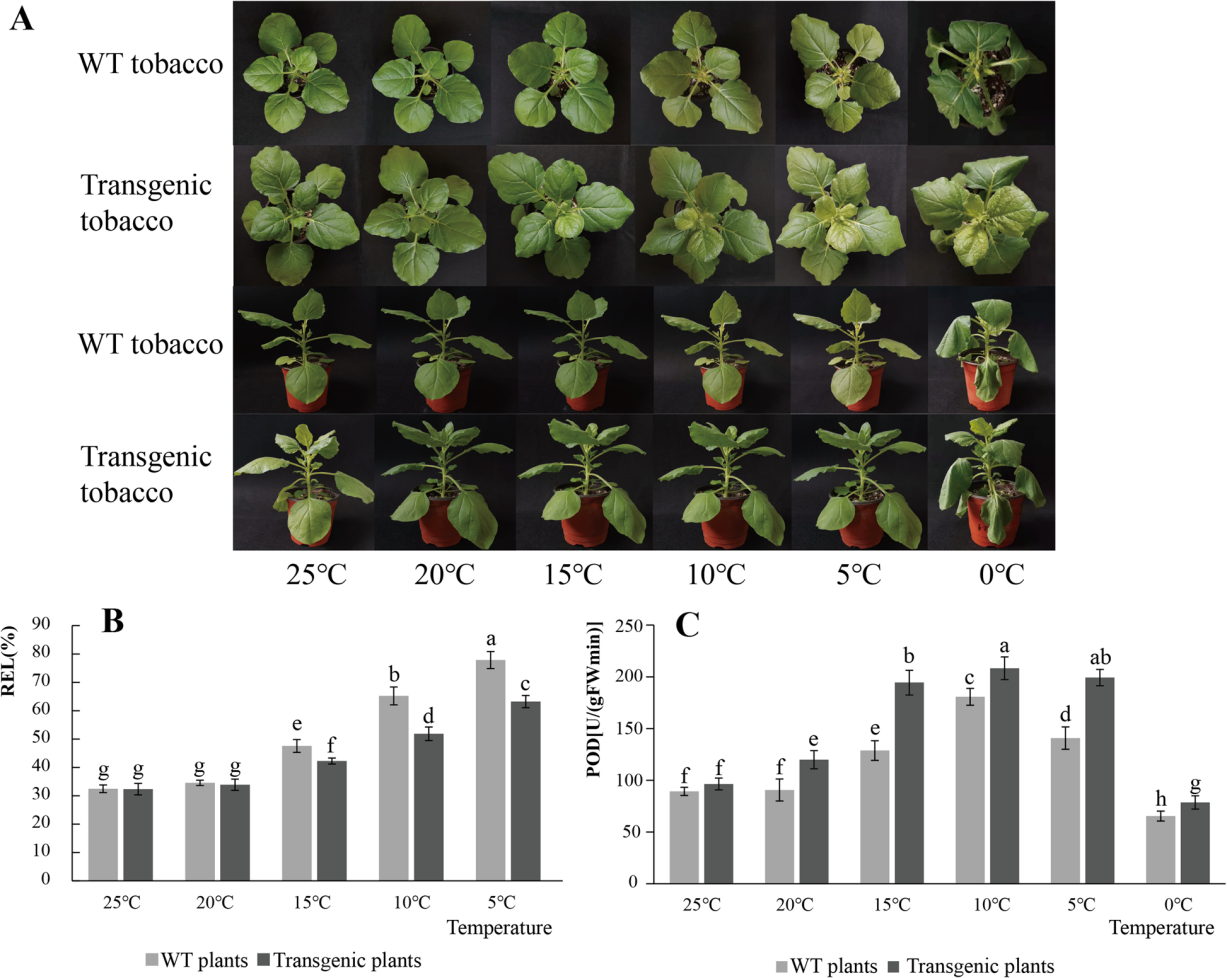
Differential analysis of wild-type (WT) and transgenic plants under cold treatment. **A** Phenotypic changes of WT and transgenic plants under cold treatment. Nine-leaf-stage plants were placed in a light incubator with a 12-h photoperiod, and cultured at 25 °C for 48 h as a control group (CK). Then, the culture temperature was lowered to 20 °C, 15 °C, 10 °C, 5 °C, 0 °C for 48 h. **B** Relative electrolyte leakage (REL) changes in WT and transgenic plants under cold treatments. **C** Peroxidase (POD) changes in WT and transgenic plants under cold treatments. Nine-leaf-stage plants were cultured at each temperature for 48 h. Columns denote the average under each temperature, and the standard errors are marked

*HfSWEET17* had the highest relative expression level in the daylily *SWEET* family, and its expression level gradually increased with the decrease in temperature. To further explore the function of *HfSWEET17* in responding to low-temperature stress, it was taken and ectopically expressed in tobacco through *Agrobacterium*-mediated transformation. Under normal conditions (25 °C), the leaf size of transgenic plants was significantly larger than those of the wild-type (WT) tobacco plants (Fig. 8A). When exposed to cold stress conditions, all lines exhibited mild cold injury, chlorosis, and slightly curled leaf margins before the temperature dropped to 5 °C, but no significant difference between transgenic and WT plants was observed. When the temperature dropped to 0 °C, all lines were wilted, but the transgenic plants showed significantly better health than the WT plants (Fig. 8A).

In normal conditions, the levels of relative electrolyte leakage (REL) and peroxidase (POD) activity were not significantly different between the WT and transgenic plants. With the decrease in temperature, the REL level of leaves from all transgenic and WT plant leaves showed an increasing trend, while the POD activity of leaves from all transgenic and WT plant leaves showed a trend of first increasing and then decreasing (Figs. 8B and C). The transgenic plants showed significantly lower REL levels by 1.13-, 1.26-, and 1.23-fold under 15 °C, 10 °C, and 5 °C, respectively, than that of WT plants. The activity of POD was significantly increased in transgenic plants compared with WT plants, and was 1.32-, 1.51-, 1.15-, 1.42-, and 1.2-fold higher at 20 °C, 15 °C, 10 °C, 5 °C, 0 °C, respectively.

## Discussion

Plant *SWEET*s play significant roles in physiological metabolism, growth, and development by regulating sugar transport and distribution [12]. They have been shown to be involved in pollen wall formation, anther dehiscence, seed development, and responses to various abiotic stresses [38–40]. Recently, *SWEET*s have been reported to play an important role in the low-temperature responses in plants including tea, cabbage, and *Poa pratensis *[24, 40, 41]. In the present study, the daylily *SWEET* gene family was identified and characterized through genome-wide analysis, and their expression patterns in different tissues and response to cold stress were investigated.

## *SWEET* gene family in daylily

This study successfully identified 19 *HfSWEET*s based on the daylily genome and named them *HfSWEET1*–*HfSWEET17* based on their homologs in *Arabidopsis* and rice (Table 1). The length of HfSWEET proteins ranged from 232 to 299 aa, which was similar to reports in other plants, such as 229–300 aa in litchi, 233–308 aa in tomato, and 234–301 aa in *G. hirsutum *[15, 19, 42]. Phylogenetic analysis divided 19 HfSWEETs into four clades (Clades I to IV) which was consistent with the results in *Arabidopsis*, *Vitis vinifera*, and *L. chinensis *[13, 19, 21]. Clade I, II, III, and IV contained four, seven, six, and two HfSWEET members in daylily, respectively (Fig. 1), which was similar to SWEET in banana and *Brassica rapa *[43, 44]. The results of the intron–exon location analysis showed that the number and distribution of the introns and exons of *HfSWEET*s were highly conserved, and most *HfSWEET*s possessed five or six exons (Fig. 3). The results of conserved motif analysis supported the phylogenetic analysis, which was consistent with results in *B. rapa* and *Medicago truncatula *[45, 46]. The *HfSWEET*s in Clades III and IV harbored conserved Motif 7, and the *HfSWEET*s in Clade III also harbored conserved Motifs 8 and 9 (Fig. 2), which suggested that they might have different functions in daylily.

Further chromosomal localization and synteny analysis showed that 18 *HfSWEET*s were unevenly distributed on 11 chromosomes of daylily, and only one *SWEET* (*HfSWEET16*) was distributed on the scaffold. Collinearity analysis showed there were segmental duplication events and tandem duplication events in the daylily *HfSWEET* gene family (Fig. 4). This suggested that *HfSWEET*s in daylily might have evolved through gene duplication. Gene duplication, including whole-genome duplication, tandem gene duplication, and segmental duplication events, can be a crucial factor for plant gene family evolution [46], and the latter two events have been suggested to represent the main causes of gene family expansion in plants [47]. Following gene duplication, duplicated gene pairs can under take different functions [48]. Combined with the above analysis of the characteristics of *HfSWEET*s, it was speculated that the expansion of *HfSWEETs* might play an important role in various gene functions of *SWEET *[48].

AtSWEET17 in *Arabidopsis* is localized in the tonoplast as a fructose-specific transporter and maintains natural changes in fructose levels [29, 30]. DsSWEET17 in *Dianthus spiculifolius* is also mainly localized in the tonoplast [32]. However, CsSWEET17 in the tea plant is localized in the plasma membrane, where CsSWEET17 and CsSWEET1a form homo/heterodimers and mediate the partitioning of sugars between the cytoplasm and the apoplast, thereby regulating plant growth and freezing tolerance [49]. In this study, the subcellular localization showed that HfSWEET17-GFP fusion protein was localized in both the tonoplast and the plasma membrane (Fig. 7).

### Expression patterns and functional diversity of *SWEETs* in daylily

Plant SWEETs are found to be involved in different sugar transporters, and *SWEET*s are differentially expressed in various tissues [50]. In this study, the expression patterns of most *HfSWEET*s were related to corresponding clades (Fig. 6A), indicating that *HfSWEET*s in the same clades possibly had tsimilar biological functions during the growth and development of daylily. *HfSWEET4a*, *HfSWEET4b*, *HfSWEET6a*, and *HfSWEET7* in Clade II showed the highest relative expression levels in flowers, suggesting they might be involved in inflorescence development. *HfSWEET1a*, *HfSWEET1b*, *HfSWEET2a*, and *HfSWEET3b* showed higher relative expression levels in leaves, suggesting that they may be involved in the transportation of photosynthetic products. In *Arabidopsis*, the expression of *AtSWEET17* in mature leaves was comparatively low [30]. Similarly, *HfSWEET17* in daylily showed higher relative expression in young leaves, but lower relative expression in mature leaves, suggesting that *HfSWEET17* might play a role in the growth of young leaves. Furthermore, *HfSWEET17* was highly expressed in roots, suggesting that it might play an important role in the roots, which was consistent with SWEET17 in *Arabidopsis *[29, 31].

The expression of *SWEET*s has been shown to change in response to chilling stress in cabbage and *M. truncatula *[41, 45]. Analyzing the expression patterns of 19 *HfSWEET*s under low-temperature treatment showed that compared with the control group (25 °C), the expression levels of all *HfSWEET*s in the low temperature (10 °C, 5 °C, 0 °C) treatment groups were changed and the relative expression levels of most *HfSWEET*s were increased (Fig. 6B), suggesting that more than one *HfSWEET* gene was responsive to low-temperature stress. The expression patterns of 19 *HfSWEET*s were different, and the relative expression of most of them was highest at 10 °C or 0 °C, suggesting that these genes might have functional redundancy.

Retained duplication genes are generally believed to be those involved in neofunctionalization, subfunctionalization, and nonfunctionalization, among which, neofunctionalizationn and subfunctionalization can lead to the differential spatial and temporal expression of duplicated genes [51]. In the present study, the expression patterns of the pairs of duplicated genes in daylily under low-temperature stress varied. For example, some duplicated genes, such as *HfSWEET4a/4b* and *HfSWEET4a/7*, were the same, whereas some duplicated genes like *HfSWEET13a/13b* and *HfSWEET14a/14b* were significantly different. These results indicated that some duplicated *HfSWEET*s were functionally similar which could have been due to nonfunctionalization during gene replication, while some duplicated *HfSWEET*s may have developed neofunctions or subfunctions and were functionally different [46, 50]. These results were consistent with results reported in litchi and apple [19, 52].

### Ectopic expression of *HfSWEET17* improved cold stress tolerance in transgenic tobacco

Studies have shown that *SWEET17* plays an important role in root development and response to various abiotic stresses [29–31, 49]. In the present study, *HfSWEET17* was highly expressed under low-temperature treatment (Fig. 6B). To further evaluate the roles of *HfSWEET17* in response to cold stress in daylily, *HfSWEET17* was transformed into tobacco. Morphological observations revealed that, the leaf size of the *HfSWEET17*-overexpressed lines was larger than those of the WT plants under non-stressed growth conditions (Fig. 8A). Previous reports have indicated the important role of *Arabidopsis* SWEET17 in the transport and utilization of fructose [29, 31]. Therefore, it was speculated that *HfSWEET17*-overexpression lines enhanced sugar transport from the source tissues to leaves, and increased the leaf size, which was consistent with the experimental results of Yao et al. [29]. Under 0 °C treatment conditions, *HfSWEET17*-overexpression plants showed significantly better growth status than the WT plants (Fig. 8A), indicating that transgenic plants were less damaged by chilling.

The analysis of physiological indices showed that the *HfSWEET17*-overexpressed tobacco exhibited lower REL and higher POD levels under cold stress compared to the WT plants. This may indicate that the transgenic plants have greater low-temperature resistance. These results showed that *HfSWEET17* in daylily positively regulated cold stress in tobacco, which was similar to the overexpression of *CsSWEET17*, which increased sugar transport in *Arabidopsis*, thus affecting germination and growth, as well as improving freezing resistance [49]. In summary, it can be speculated that *HfSWEET17* is a positive regulator of cold tolerance in daylily; it may promote nutrition and reproductive growth by transporting and utilizing sugars; it may protect against reactive oxygen species-mediated injury during osmotic stress and improve plant cold tolerance by enhancing POD activity [53, 54]. However, the biological function of this potential interaction remains to be further investigated.

## Conclusions

This study identified the *SWEET* gene family in daylily at the genome-wide level. A total of 19 *HfSWEET*s were identified and comprehensively characterized through phylogenetic analysis, conserved motif prediction, exon–intron structure, chromosomal localization, and synteny analysis. Phylogenetic analysis classified 19 *HfSWEET*s into four clades (Clades I to IV). The expression patterns of all the *HfSWEETs* in different tissues and under low temperature treatments indicate that the genes in the same clades may have similar biological functions during the growth and development of daylily and they may be involved in low-temperature stress signaling pathway regulation. Furthermore, the overexpression of *HfSWEET17* improved cold stress tolerance in transgenic tobacco. This study laid the foundation for elucidating the functions of the *HfSWEET*s in daylily in low-temperature response.

## Materials and methods

### Plant material

The daylily cultivar ‘Golden Doll’ was grown in the botanical garden of the Shanghai Institute of Technology, Shanghai, China. ‘Golden Doll’ was dormant, showed a long flowering period, and strong resistance to multiple abiotic stresses. Generally, this cultivar loses leaves in winter and develops new leaves when the temperature rises in spring. Plants used for experiments were maintained under the same integrated botanical garden management practices. Leaves were collected, immediately frozen in liquid nitrogen, and kept at − 80 °C for experiments.

### Identification and molecular cloning of the *SWEET* gene family in daylily

The *SWEET* family member domain Hidden Markov Model (MtN3_slv, PF03083.15) from the Pfam (http://pfam.xfam.org/) database [55], was used to retrieve the daylily genome database (unpublished) by HMMER3.0 and SPDE software [56, 57]. The results were sequentially sorted to remove redundancy, and candidate genes of daylily *SWEET* gene family members were preliminarily obtained. Then, the candidate sequences were identified by SMART (http://smart.embl-heidelberg.de) and NCBI-CCD (https://www.ncbi.nlm.nih.gov/cdd) [58, 59].

Total RNA was extracted from leaves using the Quick RNA Isolation Kit and the quality of the RNA was analyzed by 1.5% (w/v) agarose gel electrophoresis and NanoDrop One. The first-strand cDNA was synthesized using the M-MuLV First Strand cDNA Synthesis Kit. The coding sequences of daylily *SWEET*s were amplified from cDNA using gene-specific primers (Additional file 4: Table S3). PCR amplification was carried out using the Taq DNA Polymerase Kit in a PCR Thermal Cycler (Bio-Rad, S1000, USA). All PCR products were purified with the Prep Column PCR Product Purification Kit, and the purified PCR products were then sequenced and the consensus sequences were deposited in GenBank (Additional file 1: Table S1). All the above kits and primers were provided by Sangon, Shanghai, China.

### Sequence analyses

ProtParam (https://web.expasy.org/protparam/) was used to analyze the amino acids, molecular weights, and theoretical isoelectric points of daylily *SWEET* family members. Transmembrane domains were predicted by TMHMM Server v2.0, and the MtN3/saliva (PQ-loop repeat) domain position was searched by NCBI-CCD.

### Phylogenetic analysis

AtSWEET protein sequences in *Arabidopsis* were obtained from the TAIR database (https://www.arabidopsis.org/). Rice OsSWEET, *V. vinifera* VvSWEET, and *Zea mays* protein sequences were obtained from NCBI (https://www.ncbi.nlm.nih.gov). Clustal Omega (https://www.ebi.ac.uk/Tools/msa/clustalo/) was used to perform homologous sequence alignment of the SWEET protein sequences in daylily and other plants, and Jalview 2.10.2 software was utilized to highlight conserved or similar amino acid sequences (Additional file 2: Table S2). Based on the results of sequence alignment, a neighbor-joining phylogenetic tree was constructed by MEGA7.0 with 1000 bootstrap replicates [60].

The NCBI website was searched for homologous protein sequences to the HfSWEET17 protein sequence, and the SWEET17 protein sequences were downloaded from 24 different plants, including *Elaeis guineensis*, *Ananas comosus*, and *Mangifera indica*. Clustal Omega was used to perform homologous sequence alignment of the HfSWEET17 and SWEET17 from 24 other plants, and Jalview 2.10.2 software was utilized to highlight conserved or similar amino acid sequences. Based on the results of sequence alignment, a neighbor-joining phylogenetic tree was constructed by MEGA7.0 with 1000 bootstrap replicates.

### Gene structure analysis and prediction of conserved motifs and domains

The exon–intron structures were analyzed by GSDS (http://gsds.gao-lab.org/) [61]. MEME (http://meme-suite.org/) was used for conserved protein motif prediction, and the NCBI conserved domain database was used to predict the conserved domains of the *SWEET* family members of daylily.

### Chromosomal distribution and gene synteny analysis

The positions of daylily *SWEET*s on chromosomes were obtained from the daylily genome annotation files (unpublished). *Arabidopsis* and rice genomes were both obtained from Ensembl Plants (https://plants.ensembl.org/index.html). Furthermore, the synteny analysis among members of the daylily *SWEET* family members and the synteny analysis between daylily and *Arabidopsis* and rice were constructed using MCScanX and TBtools [62, 63].

### Expression pattern analysis of *SWEETs* in daylily

To analyze the expression patterns of *SWEET* family members in different tissues, qRT-PCR analyses were carried out on the young leaves, mature leaves, old leaves, flowers, and roots of the ‘Golden Doll’ daylily. To investigate the expression patterns of *SWEET*s under low temperature, the daylilies were moved to an indoor incubator at a constant temperature and cultured at 25 °C (control group, CK), 10 °C, 5 °C, and 0 °C with a 12-h photoperiod for 24 h. Samples were collected from mature leaves. All samples were frozen in liquid nitrogen immediately after collection and stored at − 80 °C. Primers based on the cDNA sequences of daylily *SWEET* family members were designed by Primer5 (https://sg.idtdna.com/pages/tools/primerquest) (Additional file 4: Table S3). Ubiquitin (UBQ) was used as the internal reference for qRT-PCR [64].

Total RNA was extracted from leaves using the Quick RNA Isolation Kit (Sangon, Shanghai, China), and the first-strand cDNA was synthesized using the M-MuLV First Strand cDNA Synthesis Kit (Sangon, Shanghai, China). Real-time quantitative PCR amplification was performed by AceQ qPCR SYBR Green Master Mix (Vazyme Biotech). Amplification was initiated with a denaturation step of 5 min at 95 °C, followed by 40 cycles of 95 °C for 10 s and 60 °C for 30 s. Fluorescence signals were detected at the end of every cycle. All reactions were performed using the Real-Time PCR Detection System (QuantStudio 5, USA), and data were analyzed using QuantStudio™ Design and Analysis Software. All reactions were performed in triplicate. Changes in gene expression were calculated using the 2^−ΔΔCt^ method [65]. Statistical analysis was performed by SPSS 20 software, and a one-way analysis of variance (ANOVA) was performed for the relative expression of *HfSWEET*s under different temperatures.

### Construction of *HfSWEET17* transient expression vectors and subcellular localization

The ORF of *HfSWEET17* was amplified using primers (Additional file 4: Table S3) containing the *EcoR* I/*Spe* I restriction sites, and *HfSWEET17* was inserted into the modified pC131-YFP vector framework under 35S. The primers used are listed in Additional file 4: Table S3. The recombined plasmids were then transformed into *Agrobacterium tumefaciens* strain GV3101 through shock transformation[66]. Organelle markers, including ER marker HDEL-mCherry, and nuclear localization sequence (NLS) marker pBin-NLS-mCherry, were used in co-transformation experiments with the 35S:HfSWEET17-GFP. The cultures were injected into tobacco (*Nicotiana benthamiana*), and the fluorescence distribution in leaf cells was observed under a confocal laser microscope (Leica STELLARIS 5, Germany) after 48 h dark culture. Leaves transformed with the 35S:YFP vector alone were used as controls.

### Binary vector construction and transformation of *HfSWEET17*

To construct an overexpression vector, the coding sequence of *HfSWEET17* was amplified from recombinant plasmids using primers with appended restriction sites *EcoR* I/*Pst* I (Additional file 4: Table S3), digested, purified, and then subcloned into the enzymatic digested modified pCAMBIA1301 downstream of the constitutive CaMV 35S. The constructed binary vector was transformed into GV3101. The generation of transgenic tobacco was performed following the leaf disc transformation method [67].

Transgenic plants were selected using hygromycin B (50 mg/L) and confirmed by PCR analysis. The positive plants were harvested and sown. Each generation of transgenic plants was verified by PCR to ensure that the *HfSWEET17* gene was inserted into the tobacco genome. The T3 transgenic tobacco plants and WT plants were cultivated under the same growing conditions.

Nine-leaf-stage plants were placed in a light incubator with a 12-h photoperiod, and the samples were collected after 48 h of incubation at 25 °C for the CK. Then, the culture temperature was lowered to 20 °C, 10 °C, 5 °C, 0 °C for 48 h. Fully expanded leaves were collected after each treatment. Each group was set up with six biological replicates. Then, the level of REL and the activity of POD were measured for each sample. WT tobacco plants served as the negative control, and the cold resistance of transgenic plants was analyzed.

## Supplementary Information

**Additional file 1:** Table S1 CDS sequences of 19 HfSWEET genes.

**Additional file 2:** Table S2 The protain sequences used to generate phylogenetic tree.

**Additional file 3:** Figure S1. Multiplesequence alignment of the SWEET17 from daylily (*Hemerocallis fulva*) andother plants.

**Additional file 4:** Table S3 PCR primer sequences used in this study.

## Data Availability

The datasets generated and analysed during the current study are available in the GenBank repository, GenBank accession Nos. OM264165–OM264183 and all sequences are provide in Additional file 1.

## Abbreviations

SWEETs: Sugar Will Eventually be Exported transporters
qRT-PCR: Quantitative real-time polymerase chain reaction
SUTs: Sucrose transporters
MSTs: Monosaccharide transporters
ORFs: Open reading frames
REL: Relative electrolyte leakage
POD: Peroxidase
AA: Amino acids
MW: Molecular weight
pI: Isoelectric point
II: Instability index
AI: Aliphatic index
GRAVY: Grand average of hydropathicity
THM: Prediction of the number of transmembrane helix
YFP: Yellow fluorescent protein
ER: Endoplasmic reticulum
NLS: Nuclear localization signal
WT: Wild-type
CK: Control group
