# Supplementary material for: Genome-wide identification and expression analysis of the SWEET gene family in daylily (Hemerocallis fulva) and functional analysis of HfSWEET17 in response to cold stress
Source: BMC Plant Biol. 2022 Apr 25;22:211. doi: 10.1186/s12870-022-03609-6 (PMC9036726; doi:10.1186/s12870-022-03609-6)
Supplement: Supplementary file 3 — Additional file 3: Figure S1. Multiplesequence alignment of the SWEET17 from daylily (Hemerocallis fulva) andother plants. [file 12870_2022_3609_MOESM3_ESM.docx]

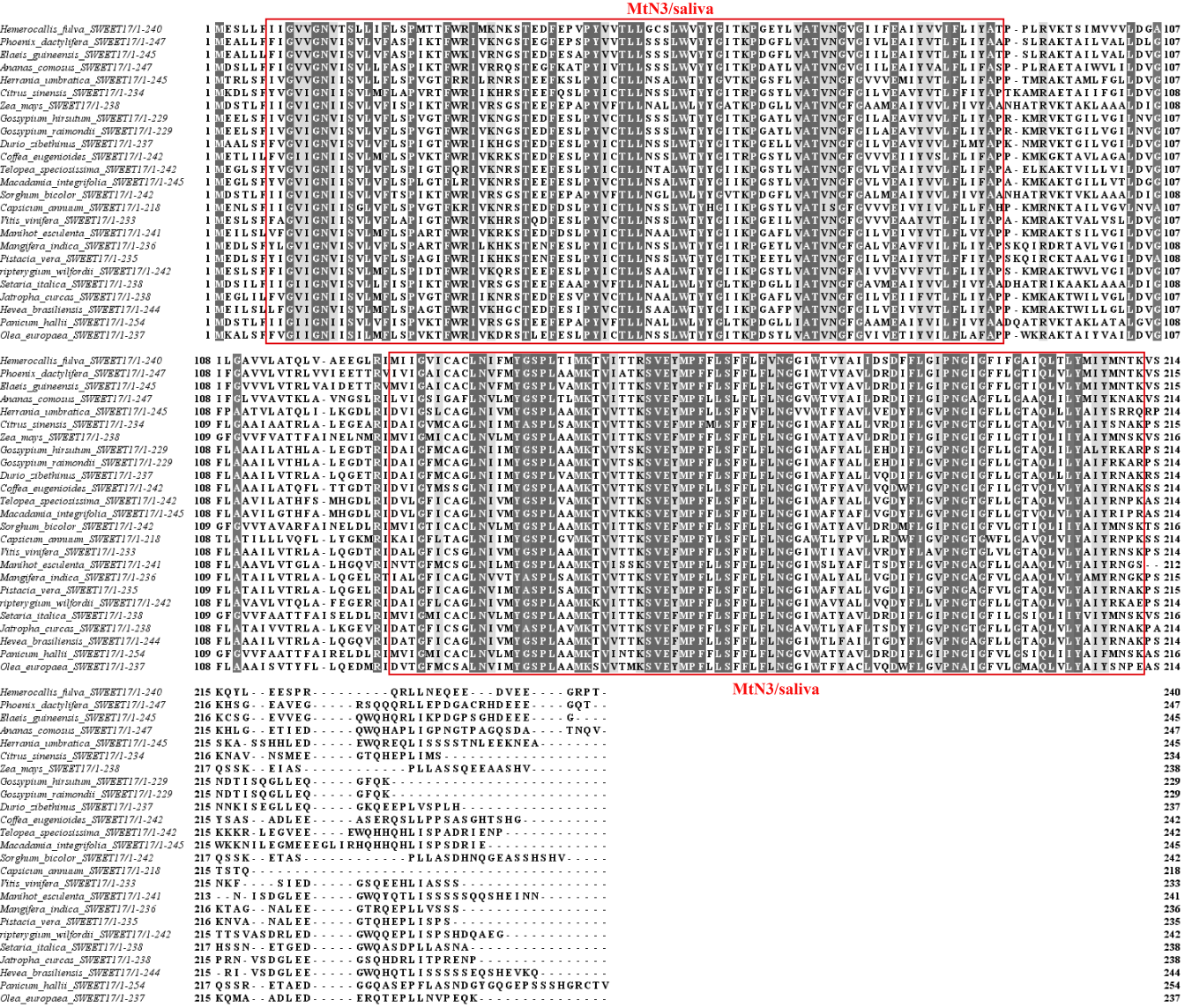


Figure S1. Multiple sequence alignment of the SWEET17 from daylily (*Hemerocallis fulva*) and other plants

The protein sequences of the SWEET17 from daylily and 24 other plants were aligned by Clustal Omega, and Jalview 2.10.2 software was utilized to highlight conserved or similar amino acid sequences. The sequences contained in the red boxs were conserved domains unique to these SWEET17.


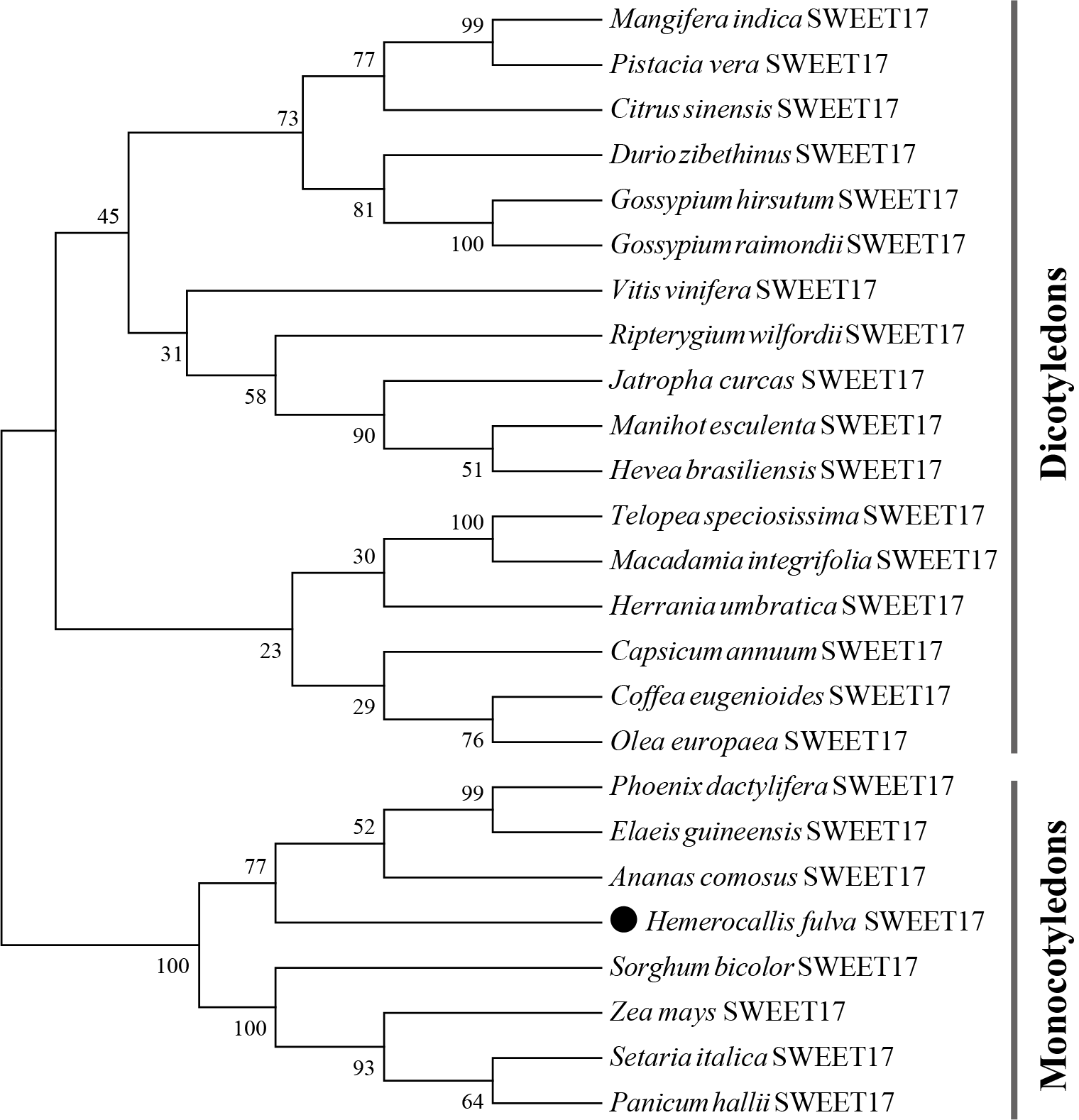


Figure S2. Phylogenetic tree of SWEET17 from daylily (*Hemerocallis fulva*) and other plants

The protein sequences of the SWEET17 from daylily and 24 other plants were aligned by Clustal Omega, and the phylogenetic tree was constructed by the MEGA7.0 using the neighbor-joining method with 1000 bootstrap replicates.


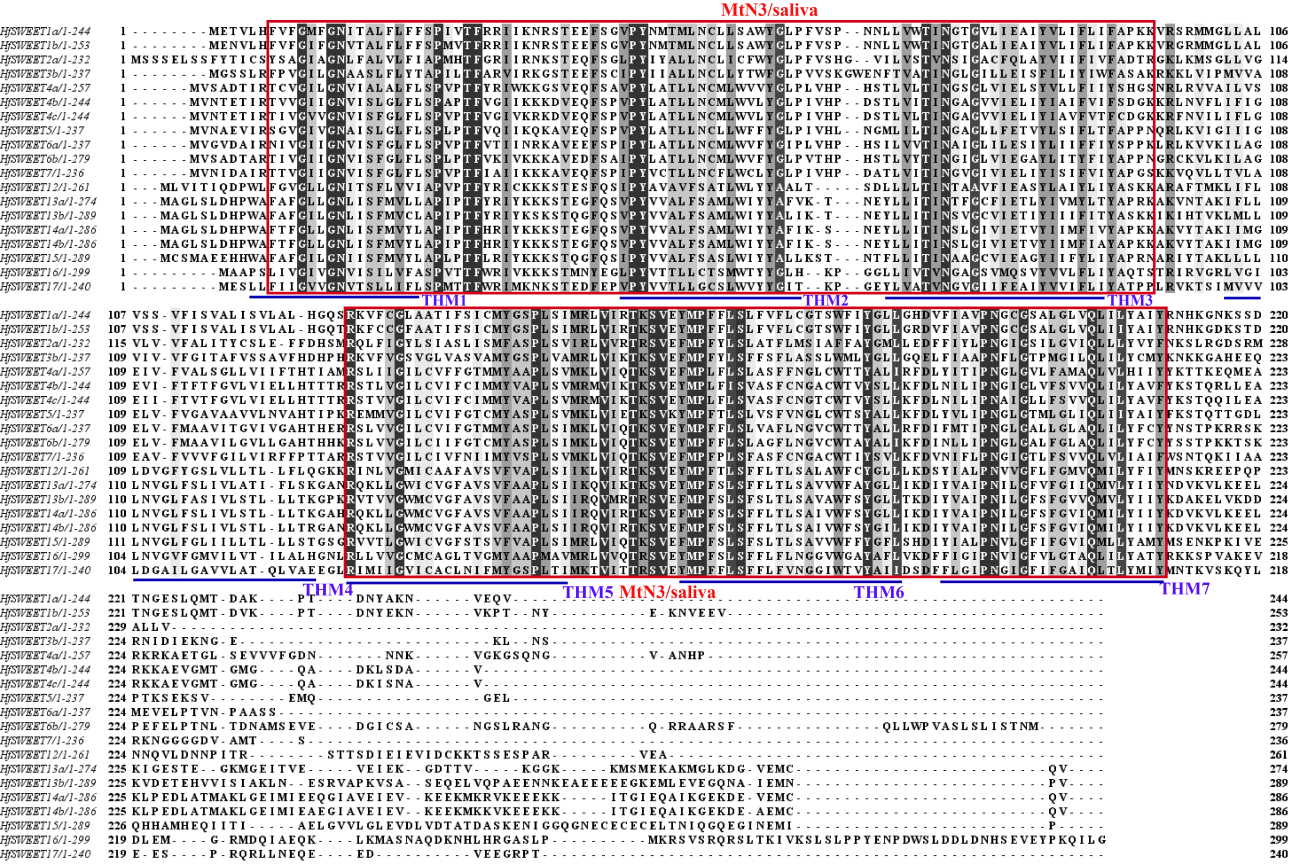


Figure S3. Multiple sequence alignment of the HfSWEETs

The protein sequences of the HfSWEETs were aligned by Clustal Omega, and Jalview 2.10.2 software was utilized to highlight conserved or similar amino acid sequences. The sequences contained in the red boxs were conserved domains unique to HfSWEETs, and the blue underlines were the positions.
